# Some Principles Relating to Amalgams

**Published:** 1895-04

**Authors:** Edward C. Kirk

**Affiliations:** Philadelphia


### SOME PRINCIPLES RELATING TO AMALGAMS.[1]

BY EDWARD C. KIRK, D.D.S., PHILADELPHIA.

The knowledge which we possess relating to dental amalgams has been achieved almost wholly as a result of the empirical method of study. The observation that filings from a silver coin, when made into a plastic mass with mercury, possessed the power of setting or hardening into a compact solid body probably first suggested the use of amalgam as a filling-material. The defects, which by experience in its use were disclosed in this amalgam, led to a further study of the subject, the object of investigation being the elimination of the defective features of the amalgam, with the hope

[1] Read before the New York Odontological Society.

of securing one that should possess certain desirable physical and chemical properties, thus raising its standard of excellence to a position more nearly approaching that evanescent vision, the ideal filling-material. In the course of time, and through the collective investigations of certainly a majority of dental practitioners, each member of the whole list of available metals, in varying proportions and relationships, has been impressed into the dental amalgam service, and made to show cause why it should not furnish the key to unlock the dental Utopia. But though each combination has been weighed in the balance of practical experience and found wanting in some essential feature, when measured by the ideal standard, still the average character of amalgam alloys has exhibited a most marked improvement, and there are now to be found and are everywhere obtainable alloys that fulfil their function as savers of teeth under certain conditions, in a manner unapproached by any other material.

As has been previously stated, this improvement of amalgams has been largely the outgrowth of the empirical, I might say "rule of thumb," method of study. No disparagement of that method is intended, nor should any reflection be cast upon it when its function is fairly recognized, for it is an essential part of the scientific method, and the basis of scientific advancement. It represents the fact-gathering stage of knowledge, and furnishes the plasma from which scientific generalization and reasoning has its growth and draws its sustenance. The element of weakness in the empirical method of study is that, unless pursued by those whose powers of observation are trained to accurate work, the resulting statement of the phenomena observed is likely to include irrelevant factors which detract from its value. This feature, unfortunately, characterizes much of the reported results of study of amalgams and their value as filling-materials. I hope to furnish concrete illustration of this point later.

A general review of the nature of the work which has been done with reference to amalgams will, I think, disclose the fact that investigation has addressed itself principally to the ingredients of the amalgam alloy, and the modifications which these ingredients, in kind, number, and amount, exert upon the physical and chemical properties of the resulting amalgam. Regarding them simply as mixtures of metals, it will be readily seen that practically an infinite number of amalgam alloys, each differing from all the others in some respect or degree, may be made with but three or four constituent elements. And further, that by the addition of mercury in variable amounts to a given alloy, still another series of amalgams, each differing from all others of the series in physical and chemical properties, may be produced. We may then, for our present purposes classify amalgams into, first, a series in which the properties of the resulting amalgam are modified by alterations in the kind, number, or amount of the ingredient elements of the alloy, and, secondly, a series in which the amalgam is modified by changes in the proportionate amount of mercury with which the alloy is combined. The classification here proposed is simply a convenience for the study of the subject. As a matter of fact, no philosophical conception of the structure, properties, and composition of alloys should admit of a separate classification for alloys without and those with mercury as a constituent. Mercury being in all respects a metallic element, its fluid state is simply a matter of relative temperature. The alloys which are products of the union of mercury with other metals are termed amalgams, simply because of the increased softness and fusibility which mercury confers upon its alloys, but these are nevertheless alloys in exactly the same sense that we apply that term to combinations of other metals into which mercury does not enter as a constituent. We may still further classify amalgams into those which are combinations of mercury with but one other metallic element, for which class I have proposed the term binary amalgams, and, secondly, those which consist of more than one metallic element in combination with mercury, which class may be termed ternary amalgams. While quite a number of binary amalgams are known, but two have had any extended use as filling-materials,—viz., copper amalgam and palladium amalgam. Silver amalgam might possibly be included in this class, but there is no record to my knowledge of the extended use of an amalgam of pure silver and mercury. The old coin-silver amalgam once used, owed its value to the small percentage of copper which it contained, and this would include it among the ternary amalgams.

In 1863, Matthiessen suggested it as probable that "an alloy is either (1) a solution of one metal in another, (2) a chemical combination, (3) a mechanical mixture, or (4) a solution or mixture of two or all of the foregoing." Investigation has served to strengthen the correctness of this view of the constitution of alloys, and dental amalgams afford some striking confirmations of it. In all amalgams which possess the quality of setting or hardening from a plastic mass, we have to deal almost certainly with a chemical combination. The property of setting is itself an evidence of chemical combination, and the formation of many amalgams is attended with elevation of temperature, more or less marked, which is another indication of chemical combination. Changes of the volume of the mass attendant upon the act of setting still further indicate that chemical union of some portion of the constituent elements of the amalgam has taken place.

There can be no doubt that the best results attainable in the formation of a dental amalgam will be those based upon the production of one in which all the constituents are chemically united in atomic ratios. Under these circumstances only have we the right to expect to produce an amalgam in which all the affinities of its constituents are satisfied, and which for that reason will not be liable to physical or chemical change. An amalgam in which all the constituent elements are not chemically united is necessarily in a condition of stress, and more subject to the influence of the chemical affinities of its environment, which perhaps slowly, but not the less surely, develop evidences of instability in the fillings made from it. While this principle has been recognized as an important and desirable one in the production of an amalgam, and though attempts have been made to practically utilize it in the preparation of amalgam fillings, the methods proposed do not seem to have achieved the object aimed at,—viz., to secure an amalgam filling in which the chemical affinities of the constituent elements are mutually satisfied. As an aid to a clearer comprehension of what it is desired to achieve in this direction, let us examine for a moment the binary combination of copper and mercury, which we know as copper amalgam. Both copper and mercury being bivalent elements, their chemical combination would naturally be made up of one atom each, and the formula of the compound would therefore be CuHg. As a matter of fact this atomic combination of mercury and copper has been found to exist. It may be made by bringing the two elements together with mercury in excess, allowing the compound to crystallize, and then squeezing out by a pressure of about sixty tons per square inch the excess of mercury. There are two other chemical compounds of mercury and copper theoretically possible, but they need not be considered here. It is only necessary to bear in mind that copper and mercury do unite chemically with each other in definite atomic proportions, that the compound is crystalline, and has the formula CuHg. It should be further noted that the atomic combination of mercury and copper, CuHg, is soluble in mercury, and that mercury may be added to it in considerable quantity without preventing its setting property or power of recrystallizing after having been softened by heat. The copper amalgams which have been used as filling-materials are probably, without exception, solutions of the atomic combination CuHg in an excess of mercury. This statement is borne out by the fact that the dental copper amalgams on the market vary considerably as to the amounts of mercury which they contain, and differ also as to the degree of heat required for their fusion. A further evidence of this feature of their structure is their behavior when heated. The gradual application of heat to dental copper amalgam causes at first an exudation of numbers of minute globules of a more fusible amalgam containing a large excess of mercury, which is followed by a softening of the whole mass. Again, when the mass has been made plastic by heat and the kneading process, and after a few minutes have been allowed to elapse to allow crystallization to commence, the excess of mercury may be squeezed out through chamois with the pliers. These phenomena in the behavior of copper amalgam furnish striking evidence that the amalgam is essentially a true chemical compound in definite atomic proportions, and that within very narrow limits any excess of mercury is an element of weakness, not only because such excess is left in a free, uncombined state which renders it more easily acted upon by the oral fluids, but because the excess impairs the integrity of the filling itself, rendering it less capable of withstanding physical wear and tear.

Knowing the chemical valence and atomic weights of the metals concerned in the formation of amalgams, it becomes a simple matter of calculation to determine the percentage amounts required in each instance to effect a combination in atomic ratios. This is especially true of the binary amalgams, but when several metals are used as constituents of an amalgam, the problem becomes exceedingly complex. I am well aware that attempts have been frequently made to accomplish the synthesis of a complex metallic compound of this character, suitable for a filling-material. The effort has resulted in various devices for adding an accurately weighed or measured quantity of mercury to as definite amount of alloy filings, but I gravely question the accuracy of the result, so far as achieving a definite chemical compound is concerned.

I regard such a method of preparing an amalgam mass as inherently bad, both theoretically and practically. While the process alluded to is evidently based upon the expectation of securing an amalgam mass in which the constituents are united in atomic ratios, there is no evidence to show that the result is attained, and, on the contrary, there are very good grounds for believing that it is not. Admitting that the components of the alloy are so related to each other and to the mercury in number and amount that, when brought together under ideally favorable conditions, a perfect atomic combination would result, we are met with practical difficulties not as yet overcome when we attempt to bring about the combination. The alloy, in the condition of shavings, filings, or coarse powder, is intimately mixed with the measured quantity of mercury, and, no matter how thoroughly the mass may be kneaded and worked, the selective affinity of the mercury will cause it to first seize upon that constituent of the alloy for which it has the strongest attraction, and thus satisfy itself before the mass is homogeneously amalgamated, or portions of the alloy will be amalgamated only superficially. The result of this will be a mass which, when set, will consist of a magma of amalgam, which is a true chemical compound, holding in its structure particles of unamalgamated alloy. Such a mass is not only lacking in homogeneity, but favors the development of local electrical action within its structure.

The desirable end of producing an amalgam in which an atomic combination of its components is secured may, I believe, be brought about in a much simpler and more practical manner by taking advantage of the selective affinity of the mercury and utilizing it for the purpose. A simple chemical illustration will perhaps make this point clearer. If to a considerable quantity of dilute hydrochloric acid we should add sodium carbonate solution in an amount not sufficient to neutralize the hydrochloric acid, we would have as the result a certain quantity of sodium chloride, formed by union of the two substances and dissolved in an excess of dilute hydrochloric acid. We could readily recover the sodium chloride from such a solution by removing the menstruum, which might be done by concentrating the liquid to the crystallizing point and separating the crystals from the mother liquor by filtration. In the process just noted, the sodium of the sodium carbonate added to the hydrochloric acid in excess has combined with only just so much of the chlorine of the hydrochloric acid as was necessary to completely satisfy their mutual affinities in the formation of sodium chloride, in accordance with the well-known chemical law that all combinations of elements take place in definite proportions by weight.

Now, we have seen that there is positive ground for the belief that amalgams, and especially those concerned in dental work, are essentially chemical compounds; it naturally follows that these combinations of mercury with the metals composing the amalgam alloy must take place in atomic ratios, and therefore in definite proportions by weight. It matters not whether the alloy be in excess or whether the mercury be in excess, the definite mercurial chemical compound is formed and its properties are modified by the excess of whichever element or elements remains over and above the amount needed to form the definite compound. We have now to consider the two methods of manipulating amalgams which are commonly employed, and their relative values, in the light of the principle which I have just endeavored to elucidate. Our objective point being to secure a definite chemical combination in atomic proportions, let us see to what extent the end is realized in each of these methods. If to a globule of mercury filings or shavings of alloy are added until a mass of proper working quality is produced, there is absolutely no guide whatever upon which the operator may depend to indicate to him the point at which the proper amount of filings has been added to exactly satisfy the chemical affinity of the quantity of mercury which has been employed. He uses his taste and judgment in producing a mass of proper working qualities. Now, taste and judgment are such extremely variable factors that they are worse than valueless as standards of scientific exactitude. Moreover, an amalgam mass of "proper working qualities" is not by any means the object for which the amalgam is being made. First, and beyond all other considerations, it is intended to produce a filling-material which shall possess the most desirable features belonging to its class. Its working quality is, or should be, a minor consideration. The method of adding filings to the mercury is subject to another feature of inaccuracy, —viz., that the filings are liable to be added in excess, so that the resulting mass is lacking in homogeneity and is liable to local electrical disturbance. It is, of course, clear that should the alloy filings be present in excess there is no possibility, from the nature of the compound, of directly getting rid of the excess of filings.

The method of weighing or otherwise measuring the amounts of mercury and filings employed, so as to secure uniformly related proportions of the constituents of an amalgam, is of course an improvement on the method just noted, but is still so lacking in accuracy as to have nothing to recommend it in comparison with the more common method of mixing amalgams, and which we will now consider,—viz., adding mercury in excess to the comminuted alloy and then removing the excess. By adding mercury in excess we have, of course, supplied all the mercury to the elements of the alloy with which they can combine.

We have also seen that these combinations of mercury with the metals take place in definite atomic proportions. The first phenomenon which we perceive on bringing together the mercury and filings is that solution of the latter has taken place. If sufficient mercury has been employed, the mass will in a moment become of a soft, buttery, or paste-like consistency, which when rubbed between the fingers or in the palm of the hand will not show the slightest trace of solid particles, for the reason that complete solution of the solid alloy has occurred. After the lapse of another short interval crystallization slowly commences, the mass thickens slightly, and when pressed carefully between the fingers emits a peculiar, softly-grating sound very much like that which may be produced by compressing a package of powdered starch. This sound is caused by the grating or rubbing of the crystals against each other, and is the analogue of what the Germans call the "zinn schrei" produced by bending a bar of pure tin in the hands. At this stage of the process we have a mixture of a definite chemical compound or compounds between mercury and the elements of the alloy in atomic proportions and crystallized, which is dissolved in its menstruum, the excess of mercury.

As it is our purpose to utilize only the chemical compound of mercury and the alloy as a filling-material, the next step is to get rid of the excess of mercury. This up to a certain point is readily accomplished by straining out the crystals through chamois-skin by compression with heavy pliers. All of the excess of mercury, however, cannot be removed in this way and the mass left in suitable working condition. It has been proposed, and the plan is pursued by some, to use portions of the mass soft or plastic, and to add to this other portions from which more of the mercury has been removed by greater pressure. This method is open to the objection that it does not remove sufficient of the mercury,—*i.e.*, all that may safely be removed without endangering the integrity of the chemical compound of mercury and the alloy. The best method within my knowledge, and one which gives results superior to anything I have ever seen in the quality of the fillings produced by it, is to absorb the excess of mercury from the surface of the filling by means of crystal or sponge gold. The amalgam mass, after crystallization has fully commenced, should be squeezed in chamois until an easily workable mass is produced. The amalgam is then introduced into the cavity of decay and the filling built more than flush or full contour. Pellets of freshly annealed sponge gold are then rubbed into close contact with the amalgam surface by suitably-shaped instruments. This process is continued as long as any whitening of the gold will take place by contact with the filling. When no more mercury can be extracted in this way, the filling may be carefully burnished, and in a very short time—depending more or less upon the kind of alloy used—may be given a final polish.

A number of analogous processes have been advocated and are in use by various members of the profession for removing or extracting the excess of mercury from fillings. Dr. Bonwill has, I think, advocated the use of bibulous paper in packing the amalgam to "absorb" the excess of mercury. Bibulous paper will, of course, not absorb mercury, but, used as recommended by Dr. Bonwill, it compresses the mass of amalgam crystals in the filling and causes the mercury to exude upon the surface, where it may be wiped away or absorbed by sponge gold. Gutta-percha, chamois-skin, leather, rubber dam, or a round soft-rubber point will produce precisely the same effect in compressing the mass of crystals and causing the mercury to exude from the surface. None of these expedients, however, remove it. Gold-foil in the thinner numbers, as advocated by Dr. Rhein and Dr. Ottolengui, will extract the excess of mercury from a filling almost completely, but the process is tedious and unsatisfactory in comparison with the use of annealed sponge gold. I should also consider the permanent attachment of the gold-foil as a part of the filling an objectionable feature of this method. Tin-foil and shredded tin have been used for the same purpose with fairly good results, but preference should be given to gold in this connection on account of its superior affinity for mercury.

Reference was made in the early part of this paper to the empirical method of observation and its proper status in comparison with the scientific method. The various procedures which have been cited relating to the extraction of the excess of mercury from amalgam fillings furnish the concrete example referred to of the inherent weakness of the empirical method as a sole means of arriving at the truth. It has been a matter of common observation that fillings of amalgam made with a minimum amount of mercury gave better results than those in which mercury was in excessive amount. Hence various devices in removing or getting rid of the excess of mercury have been brought out. Both the observation and the devices are largely of the empirical order, and each of the latter has its advocates. It would seem, however, with the mass of data at our command that we should have some better reason for the use of a given method of this character than that it gives good results. We should be able to say why it gives good results, and to know, for example, whether it is best to mix an amalgam by this or that process, whether it is better to use bibulous paper, rubber dam, gutta-percha, chamois-skin, or sponge gold to get rid of the excess of mercury, and the reasons therefor.

I have presented for your consideration in a somewhat elementary way a subject upon which, I presume, every dentist has an opinion. It is this very multiplicity of opinions which, unless properly controlled and related by scientific method, will greatly inhibit the usefulness and proper application of the material under consideration. Our results, save a few notable exceptions, have been too much the product of guesswork and imperfect methods of investigation. We cannot secure the highest results in this, or, in fact, in any of our departments until we conduct our observations and investigations and formulate our results in harmony with the only logical method of reasoning, the scientific method.

